# A Kettle of Fish: A Review of the Scientific Literature for Evidence of Fish Sentience

**DOI:** 10.3390/ani12091182

**Published:** 2022-05-05

**Authors:** Helen Lambert, Amelia Cornish, Angie Elwin, Neil D’Cruze

**Affiliations:** 1 Animal Welfare Consultancy, Kingsteignton TQ12 3BW, UK; 2 Independent Animal Welfare Consultant, Caulfield South 3162, Australia; milliecornish@googlemail.com; 3 World Animal Protection, 222 Gray’s Inn Rd, London WC1X 8HB, UK; angieelwin@worldanimalprotection.org (A.E.); neildcruze@worldanimalprotection.org (N.D.)

**Keywords:** animal welfare, emotion, fish, sentience, wildlife trade

## Abstract

**Simple Summary:**

Fish are traded, caught, farmed, and killed in their trillions every year around the world, yet their welfare is often neglected and their capacity for feelings is regularly disregarded. We have searched the scientific literature to determine what is known about fish sentience and relate our findings to the many uses of fish around the globe.

**Abstract:**

Fish are traded, caught, farmed, and killed in their trillions every year around the world, yet their welfare is often neglected and their sentience regularly disregarded. In this review, we have sought to (1) catalogue the extent to which fish sentience has featured over the past 31 years in the scientific literature and (2) discuss the importance of fish sentience in relation to their commercial uses. We searched the journal database Science Direct using 42 keywords that describe traits or elements of sentience to find articles that were referring to or exploring fish sentience. Our review returned 470 results for fish sentience in 142 different species and subspecies of fish, and featured 19 different sentience keywords. The top four keywords were; ‘stress’ (psychological) (*n* = 216, 45.9% of total results), ‘anxiety’ (*n* = 144, 30.6%), ‘fear’ (*n* = 46, 9.7%), and ‘pain’ (*n* = 27, 5.7%). Our findings highlight an abundance of evidence for fish sentience in the published scientific literature. We conclude that legislation governing the treatment of fish and attitudes towards their welfare require scrutiny so that their welfare can be safeguarded across the globe.

## 1. Introduction

Fish are experimented upon, traded, caught, and farmed in numbers far outweighing any other vertebrate taxa [1]. Currently, fish represent over half of the vertebrate species in the wildlife trade and had an estimated worth of US$180 billion in 2018 [1,2]. The commercial fishing sector is so vast that fish harvested from the wild are typically recorded by weight, rather than numbers of individuals [3]. Records generally do not account for the numbers of fish that are caught as by-catch or in ghost fishing gear [4]. Animals caught in abandoned fishing gear or in fishing nets as by-catch can suffer extensive injuries and slow deaths as a result [5,6,7].

Although most fish involved in the wildlife trade are destined for consumption (both for subsistence and commercial use), many are also caught, farmed, and traded as pets, an industry that has seen significant growth in recent years [2,8]. Figures regarding the scale of the fish pet trade remain unclear, as much is unmonitored and unregulated, and few scientific analyses have attempted to quantify the numbers involved [2]. Fish are also increasingly used for scientific research. The past 20 years, for example, have seen a steep rise in the number of zebrafish (*Danio rerio*) used as models for various human diseases e.g., [9,10,11] for developing pharmaceutical drugs [12], and in aquaculture nutrition research e.g., [13]. In addition, fish are used in tourism, including ray petting and sport fishing, where they may be subject to a range of welfare issues [14,15,16]. Fish are also traded for traditional medicines and belief-based use [17,18]. Seahorses, for example, are one of the most widely traded taxa for traditional medicines [19], with thousands of metric tonnes of their bodies traded into and out of China for this purpose each year [20]. Aquaculture has also grown exponentially in terms of the numbers of fish involved [21], and although domesticated species of fish are typically used, wild fish are also involved, as new species are continually being targeted for farming. Millions of tonnes of fish are also wild-caught to feed farmed fish and domesticated livestock every year [21,22].

Despite their wide uses in the wildlife trade, legislation protecting fish has been described by some researchers as inadequate, including for experimentation, trade, and aquaculture [23,24,25]. Nevertheless, some efforts to develop protocols for fish welfare are being seen in response to the increasing use of fish in translational research and aquaculture [26]. The disparity still seen in legislation, however, is in part due to doubt and ignorance regarding the capacity of fish to feel pain and suffer [27,28], and many consumers and policymakers still consider fish to have little capacity for subjective experience [23,28,29]. For example, fish often score low on belief in animal minds surveys compared with other vertebrates such as mammals and birds [30,31]. The capacity of fish to feel pain has been questioned historically, potentially because there are significant economic implications associated with acknowledging this [32]. Specifically, some argue that the entire commercial fishing model is based upon the idea that fish do not suffer and therefore do not need to be slaughtered, but can be left to asphyxiate instead [33,34].

Inadequate protection for fish applies to both welfare and conservation concerns, as fish and other less charismatic taxa, such as invertebrates and amphibians, have not always received the same level of attention from policy forums as other vertebrates have. For example, the Convention on International Trade in Endangered Species of Wild Fauna and Flora (CITES) currently lists only 16 species of fish in Appendix I, despite 15,000 species being traded globally, around 20% of which are threatened with extinction [35,36].

In this review, we have sought to address these issues by examining the scientific literature to quantify the extent to which fish sentience has been recognised, utilised, or experimentally explored. For the purpose of this review, we define sentience as the capacity to have feelings, but have used a list of 42 different keywords to encompass all aspects of sentience. In this review, we have (1) catalogued the extent to which fish sentience featured over the past 31 years in the scientific journal database Science Direct, both in terms of sentience traits and the species involved, and (2) discussed the findings concerning the extensive involvement of fish in the various industries that impact them.

## 2. Methods

We searched the scientific literature for evidence of sentience in fish. As the term sentience is complex, and the traits and characteristics of sentience may manifest themselves in different ways, we used a list of 42 keywords to define sentience (Supplementary File S1). The keywords were derived from previous reviews we have performed exploring sentience in all animals [37], reptiles [38], insects [39], and amphibians [40]. Because of the scale of this current review, we only searched for the keywords that had returned results in the previous four reviews. We also added two additional keywords, namely ‘intentionality’ and ‘prosocial’, to encapsulate additional aspects of sentience that were not used in the previous reviews.

We used the 42 keywords to search through the journal database Science Direct. We performed the searches using the search term ‘fish’, along with the Boolean operator ‘AND’, and then each of the keywords in turn. As some fish genera are not commonly referred to as fish and would therefore be unlikely to come up when searching for ‘fish’, we performed additional searches to ensure that we covered as many species of fish as possible. Therefore, we also searched for ‘seahorse’, ‘eel’, ‘shark’, and ‘ray’, along with each of the sentience keywords. We performed 210 searches in total.

Each of the articles was reviewed against a set of criteria. Those that met all five of the criteria were included in the results as a returned article. The criteria were that the article (1) had to be published between 1990 and 2020, (2) be a research article, (3) utilise one or more species of fish in an experiment, (4) use the keyword regarding the fish species being studied in their experiment and not a previous study, and (5) use the keyword in relation to the fish’s subjective state. For example, the keyword ‘stress’ had to be used regarding the emotional or subjective experience of stress, and not oxidative or thermal stress. Some articles referred to more than one keyword or species, and we treated these as individual results, providing they met the criteria. We focused on the timeframe of 1990–2020 in order to cover a large selection of research within the time constraints that we had. The research was performed in 2021 to ensure that 2020 was a complete year.

We reviewed all the results to establish how the keyword was used regarding the fish species. Each returned result was categorised as either ‘assume’ or ‘explore’, depending on whether the keyword was accepted or being experimentally explored. For example, a study that ‘assumed’ that parrotfish can experience pleasure would use the keyword in their experiment to test whether parrotfish found one thing more pleasurable than another, whereas a study that was ‘exploring’ whether fish can feel grief would test to see whether they showed behavioural and physiological signs of grief in an experimental paradigm. We recorded the ‘explore’ results as either positive, negative, or inconclusive, depending on the study’s authors’ reported outcome. If they found positive evidence of the keyword they were exploring, then we recorded it as a positive result. If they found evidence that the fish species did not have the characteristic or trait they were exploring, then we classed it as negative. We recorded ambiguous findings as inconclusive.

We then recorded the article’s title, publication year, and journal name for each result, along with the scientific name and common name of each of the relevant species, and their taxonomic order(s). To maintain consistency, following the initial searches, one researcher reviewed all the returned articles against the criteria to determine which articles were to be included in the review.

### 2.1. Additional Criteria

In addition to the five criteria mentioned earlier, some keywords were subject to additional criteria due to their use. The keywords ‘stress’, ‘distress’, and ‘suffer’ were commonly mentioned in relation to ethical procedures or regulations that the study had to adhere to. For these keywords, we only included the article if the keyword was used directly about the fish species being studied and not used only as a sweeping statement. For example, if the study stated that ‘care was taken to reduce distress in the zebrafish’, then it was included as it referred specifically to the fish species. However, if it stated that ‘care was taken to reduce distress in the animals used in the experiment’, this phrase was considered to be too general, as it did not refer specifically to the fish species used and, therefore, did not fit the criteria for inclusion.

Science Direct caps the results it displays to a maximum of 6000. The keyword ‘stress’ was the only one to return over 6000 results (92,695 results). Therefore, we could only view the first 6000 of these results (organised by relevance).

### 2.2. Inter-Rater Reliability Tests

Two of the authors collected the data, and both had previously performed a similar systematic review using many of the same keywords [37]. Both researchers conducted two inter-rater reliability tests [41] before and after the data collection period. For each of these tests, both researchers reviewed the same six articles and recorded whether the keyword was used correctly for each and whether it was explored or assumed. Each test used three randomly selected keywords, and a different selection of six articles was used for each test. The lead researcher’s analyses served as the gold standard throughout training and for all comparisons. We then compared the researchers’ responses with each another and calculated a percent agreement. Both tests returned a 100% agreement score.

### 2.3. Data Analysis

We performed descriptive analyses (totals and percentages) on the returned results using Microsoft Excel Version 2110.

## 3. Results

In total, 470 results were returned across 349 articles. Some articles mentioned more than one keyword or utilised more than one species of fish, and we recorded those as individual results.

### 3.1. Sentience Keywords

**Figure 1:**
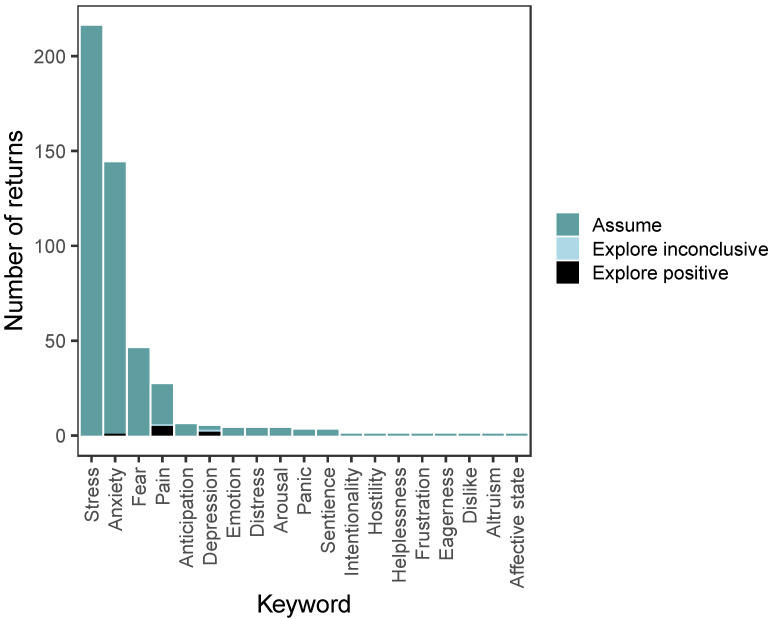
The number of studies returned for each of the sentience keywords we searched for in our search of the literature for evidence of sentience in fish. The graph shows the number of times each keyword was studied in fish across the 349 returned articles published between 1990 and 2020, and whether the keyword was accepted in the study (‘assume’) or was being experimentally explored.

Of the 42 sentience keywords we searched for, 19 returned results (see Figure 1). The top four keywords returned were ‘stress’ (*n* = 216, 45.9% of total results), ‘anxiety’ (*n* = 144, 30.6%), ‘fear’ (*n* = 46, 9.7%), and ‘pain’ (*n* = 27, 5.7%).

### 3.2. Taxa Returned

Of the 470 results, 75.3% (*n* = 354) were returned using the search term ‘fish’ (along with each of the sentience keywords), 15.1% (*n* = 71) were returned using the search term ‘shark’, 1.7% (*n* = 8) were returned using the search term ‘seahorse’, and 0.6% (*n* = 3) were returned using the search term ‘eel’. The search term ‘ray’ did not return any appropriate results.

**Figure 2:**
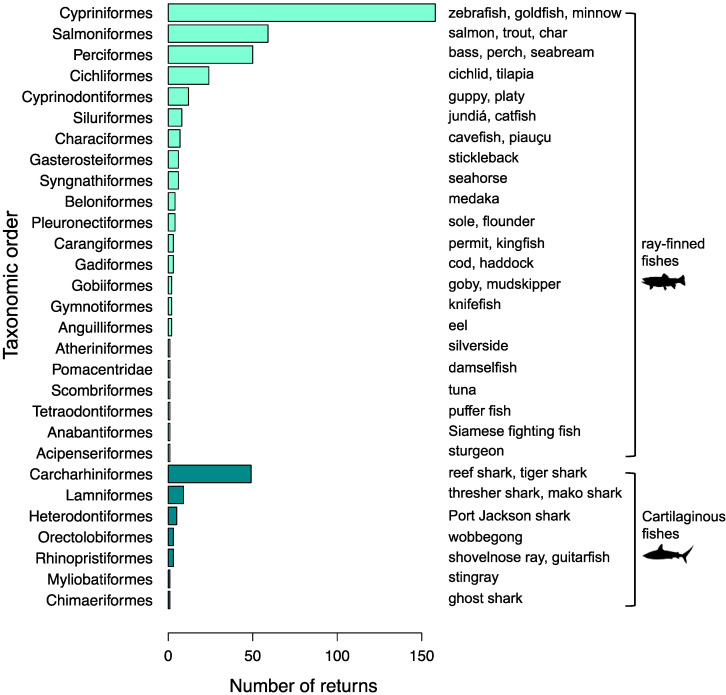
The number of returns from our search of the literature for evidence of sentience in fish. The graph shows the number of times each order featured in studies published between 1990 and 2020 that either assumed or explored sentience in fish, along with the most frequently returned types of fish that featured for each order.

In total, 142 different species and subspecies of fish were returned (Supplementary File S2), representing 30 different fish orders (see Figure 2). The top three orders returned were Cypriniformes (ray-finned fish such as carp and zebrafish; *n* = 185, 39.3% of all results), Salmoniformes (ray-finned fish such as salmon and trout; *n* = 60, 12.9%), and Perciformes (ray-finned fish such as perch and cichlids; *n* = 51, 10.8%). The top three species featured were zebrafish (*Danio rerio*, *n* = 120, 25.5% of all results), rainbow trout (*Oncorhynchus mykiss, n* = 45, 9.5%), and Nile tilapia (*Oreochromis niloticus, n* = 15, 3.1%).

### 3.3. Explore/Assume

Only the keywords ‘anxiety’, ‘depression’, and ‘pain’ were explored (see Figure 1). ‘Anxiety’ was explored once with a positive outcome involving piauçu (*Leporinus macrocephalus*). ‘Depression’ was explored three times, with two positive results, both involving zebrafish (*Danio rerio*), and one inconclusive result involving zebrafish. ‘Pain’ was explored six times with five positive results involving common carp (*Cyprinus carpio*), goldfish *(n* = 2 *Carassius auratus*), and rainbow trout (*Oncorhynchus mykiss*), and one inconclusive result involving zebrafish. The remaining keywords were all assumed.

### 3.4. Journals

The 349 returned articles were published in 64 different journals in the Science Direct database. The three journals featuring the most articles were *Aquaculture* (*n* = 41, 11.7% of all articles), *Behavioural Brain Research* (*n* = 37, 10.6%), and *Physiology and Behavior* (*n* = 27, 7.7%).

### 3.5. Publication Years

**Figure 3:**
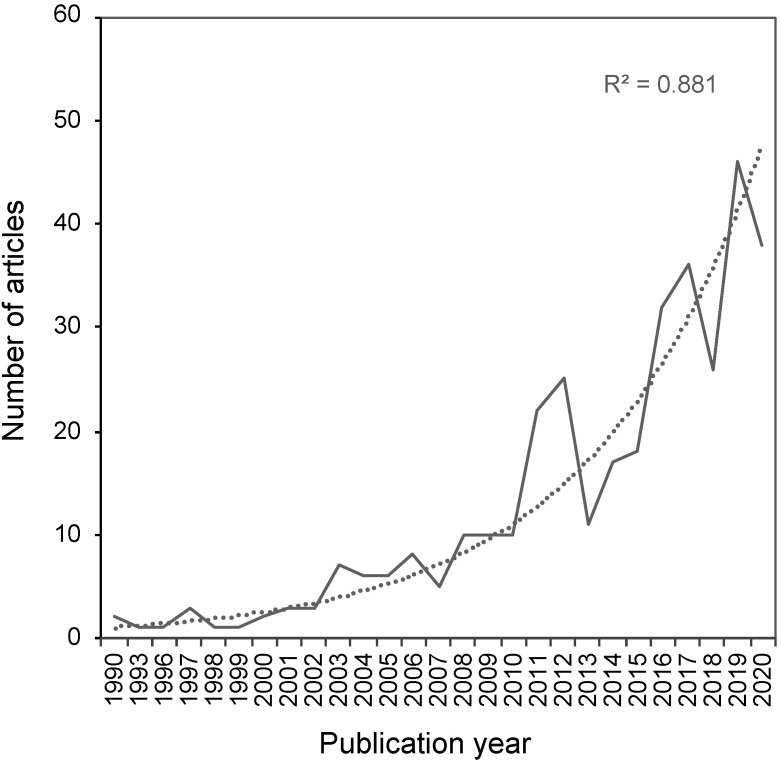
The number of articles returned from our search of the literature for evidence of sentience in fish. The graph shows the exponential growth trend (R^2^ = 0.881) in the number of research articles published each year between 1990 and 2020 that utilized one or more species of fish in an experimental study of sentience.

Figure 3 shows the number of returned articles from each of the publication years reviewed (1990–2020). Overall, there was an increasing trend (R^2^ = 0.881) in the number of articles featuring fish and the sentience keywords being published each year.

## 4. Discussion

Despite the common misconception that fish cannot feel pain or that their feelings do not matter, there is plenty of evidence demonstrating the importance of considering fish sentience e.g., [25,42,43,44]. In this review, we found evidence of fish sentience across the scientific literature, and that fish are commonly recognised as being capable of experiencing a range of emotional states. Specifically, we found 470 references to sentience traits in fish across 349 different articles published between 1990 and 2020. The results involved 142 different species and subspecies of fish (from 30 different taxonomic orders) and spanned across 19 different sentience keywords, representing a range of subjective states in fish, from altruism to stress. Of the 10 results (eight articles) that explored sentience in fish, eight positively identified evidence of pain, depression, and anxiety; the other two were inconclusive (for pain and depression). Our review has highlighted the considerable number of studies that have utilised aspects of fish sentience in experiments. Fish sentience is a growing field of research, as we found a positive exponential growth trend (R^2^ = 0.881) in the number of scientific studies that have assumed or explored fish sentience over the past three decades.

It is important to note that the majority of the results returned in our review were ‘assuming’ sentience traits in the fish species being studied. We do not feel that this is a concern in regards to evidence of fish sentience being unproven, as the same bias was found across other taxa, including mammals, in our previous review [37]. Furthermore, due to the limited scope of our review, we cannot say whether or not these keywords have previously been explored elsewhere in fish species, or what the assumption of the sentience trait was based upon. It is highly likely that, as with other vertebrate taxa such as mammals, the basis for these ‘assumptions’ comes from a mix of empirical evidence, behavioural responses, and the physiological and neurological indicators underlying the sentience trait in question.

Most of the results (75.4%) were returned when searching for ‘fish’, although some groups of fish were not featured in these searches; none of the results returned under the ‘eel’, ‘shark’, ‘seahorse’, or ‘ray’ searches were recorded in the generic ‘fish’ searches. The search term ‘ray’ returned no appropriate results, whereas ‘seahorse’ returned eight, ‘shark’ returned 71, and ‘eel’ returned three results, all of which were ‘assumed’. Overall, we found no evidence of an absence of sentience in fish. Instead, our findings highlight the capacity of fish to feel important subjective states, such as pain, depression, and anxiety, and positive evidence of the capacity of fish to experience these states. As research shows that fish are capable of a range of emotional states and feelings such as pain [28,41,44], our findings have important implications for fish, both wild-caught or farmed for use as pets, scientific purposes, traditional medicine, or consumption, as the associated welfare issues can be considerable and deserve attention [23,25,45].

### 4.1. Welfare Implications

#### 4.1.1. Fish

The sentience keywords ‘stress’, ‘anxiety’, ‘fear’, and ‘pain’ returned the most results, both when searching for ‘fish’ and overall (see Figure 1). This is unsurprising, given that fish, particularly zebrafish, are regularly used as models of anxiety and stress [46]. Given that some researchers question whether or not fish feel pain, e.g., [47], it is interesting to see that ‘pain’ was assumed in fish 21 times, including once for eels [48], in 20 different studies and in eight different fish species. ‘Pain’ was explored in six cases, five of which had a positive result and concluded that the species studied (common carp, rainbow trout, and goldfish) could feel pain. One study had an inconclusive result [49], as they did not have definitive evidence for pain in zebrafish. However, they did have a positive result for pain in common carp and rainbow trout.

Other keywords returned in our review included ‘affective state’, ‘emotion’, and ‘sentience’, all of which were assumed in European minnows (*Phoxinus phoxinus*), rainbow trout (*Oncorhynchus mykiss*), seabream (*Sparus aurata*), zebrafish (*Danio rerio*), and convict cichlids (*Amatitlania nigrofasciata*). In one study, both ‘emotion’ and ‘sentience’ were assumed in rainbow trout and were used as the basis for designing welfare measures for aquaculture [50]. In the study, the fish that were subjected to poor water quality (the stressed group) were less able to cope with aversive experiences (such as social isolation and human presence), showed spontaneous behavioural differences, and had diminished cognitive abilities compared with the control group. These findings have considerable implications for the many uses of fish. The water conditions for some commonly farmed fish species, for example, are often considered to be poor due to overcrowding and inadequate husbandry measures [51]. Similar issues are found for fish transported for the pet trade, as the duration of time they are kept in small containers can negatively impact water quality and severely restrict their natural behaviour [8].

#### 4.1.2. Sharks

‘Stress’ was the only keyword that returned suitable results for sharks, and all 71 results were assumed across the 40 species featured (Supplementary File S2). Sharks are affected directly by the fishing industry, as they are targeted for their fins [52] and meat [52], and are often caught in nets as by-catch [53]. Mortality rates for sharks released after being caught as by-catch are typically high, which is thought to be due to stress caused by the experience [54]. One study we reviewed examined the post-release survival and behaviour of juvenile tiger sharks when caught in long-line fishing gear [55]. They found that the stressfulness of the experience resulted in behavioural changes in sharks, and suggested that the subsequent deep-diving that the sharks performed for the days after release was a direct response to the stress caused by being hooked, and that the sharks were deliberately avoiding the shallower waters.

#### 4.1.3. Rays

Our searches for ‘ray’ returned no suitable results. Rays are often caught as by-catch; fished for the pet trade, consumption, and recreation; and for their skins [56,57]. Some species of rays, such as stingrays, are also used in research [58]. However, there is very little understanding regarding what constitutes good welfare for rays during capture or captivity. To our knowledge, no studies have explored the welfare of rays kept as exotic pets or in the preceding stages of trade, and it appears there are no strategies in place to ensure their welfare during commercial fishing, despite the apparent rise in interest in their skin for leather products, their meat, and the capture of live rays for the exotic pet trade [56,59,60]. Stingrays are becoming increasingly popular pets, and some species are sold for over USD 500 [61]. As they are unsuited to home aquariums, owners often release their stingrays into waterways once they outgrow their captive environment, which can pose a risk to native aquatic species [59,61]. Rays also feature in public aquariums, where people can stroke and interact with them. Despite this, we found no reference in the scientific literature on their welfare during these interactive experiences.

#### 4.1.4. Eels

‘Pain’ and ‘stress’ were the only keywords that returned results for eels, both of which were assumed in the two studies (pain: *n* = 1; stress: *n* = 2), and all three results featured the European eel (*Anguilla anguilla*). One study concerned the welfare implications of live chilling and freezing of farmed eels, and tested the time it took eels to reach unconsciousness, based upon ECGs and their pain responses [48]. Eels are both farmed and wild-caught for consumption and breeding, and may be exposed to many stressful and painful experiences, including injuries and mortalities as a result [62]. Little is known scientifically about the subjective experiences of eels, but evidence of their capacity to feel pain and stress means that their welfare deserves more attention.

#### 4.1.5. Seahorses

Our results show that seahorses are assumed to be capable of anxiety, distress, fear, and stress, which are all highly relevant emotional states in terms of their use in the pet trade, in scientific research, and as traditional medicine. The main two markets for seahorses are the trade of dried seahorses for traditional Asian medicine [20], which comprised an estimated 5.7 million individuals between 2004 and 2011 [63], and the trade of thousands of live seahorses for the pet trade [64]. There is currently very little in the scientific literature regarding the welfare of seahorses in both of these markets. Therefore, more research is needed, particularly when considering the extent of their trade and that they are assumed in research to have the capacity for emotional states such as distress and fear. In one study we reviewed, lined seahorses (*Hippocampus erectus*) showed both behavioural and physiological stress and distress responses to chronic noise exposure, compared with when they were kept in a quiet tank [65]. These findings have direct consequences for the welfare of the thousands of individual seahorses traded as pets, as the transportation and subsequent captivity may well involve exposure to chronic noise, representing a significant welfare concern.

### 4.2. Limitations

Our literature review was not intended to be exhaustive, but highlighted what scientists accept and are actively researching regarding fish sentience. Given the broad scope of the terms that we used (fish, seahorse, eel, shark, and ray) and the fact that we searched the full text of the articles, it is likely that we found the vast majority of relevant Science Direct articles, although we may still have missed some studies.

The sentience keyword ‘stress’ returned over 90,000 research articles. Unfortunately, because Science Direct only allows one to access the first 6000 articles (organised by relevance), we could only review and include the relevant articles from the first 6000, rather than the full cohort of studies referring to stress. As a result, it is likely that the total number of returned articles and results for the keyword ‘stress’ are far higher than we have found.

### 4.3. Future Research

Given that there are around 32,000 extant species of fish [23], adopting a species-specific approach for reviews such as this is unfeasible. Future reviews and research into fish sentience could focus on species that are utilised, traded, farmed, or caught in extensive numbers to develop our understanding of the complex welfare needs of fish, and the relevance and application of these to their use in various industries. For example, stingrays are growing in popularity as pets [59] and are sought after for their skins [56], and eels are widely caught and farmed for consumption [48]. However, our review found little research into the subjective minds of eels and rays, and so more research into their welfare needs is important.

Our review primarily returned sentience keywords that are concerned with negative states and experiences such as pain, stress, and fear. Animal welfare science increasingly recognises the importance of positive states and experiences for captive animals [66]. Our review highlights that there is currently a lack of either acknowledgement or utilisation of positive states in fish in scientific research. This may be because many do not consider fish as capable of positive experiences or states such as joy, pleasure, and play, or it may be because these keywords were not relevant to the types of research that fish are commonly used for.

Discussions and research into fish welfare are still very much lacking, especially compared with other vertebrates [24,37]. Even with the rise of aquaculture, progress has been criticised as being ‘exceptionally slow’ in addressing major welfare issues such as slaughter, handling, disease, and stocking densities [23]. It is unsurprising, therefore, that positive experiences and feelings in fish are relatively unexplored, as they have only recently become a concern for other vertebrates in the past decade [37,66]. However, if we are to maximise the welfare of fish in laboratories, home aquariums, or aquaculture facilities, then more research is needed into both positive and negative experiences in fish, how they affect fish welfare, and what fish need to have a good life.

Another important direction for future research should be on how to improve public attitudes to and perceptions about fish. Fish are commonly viewed merely as commodities and are typically described in terms of volume or weight, rather than the numbers of individual fish who are bred, caught, and killed [3,24,27]. Pet fish are also commonly viewed as disposable and are often simply replaced when they are ill, rather than treated [67,68]. As a result, the treatment of fish in many contexts can be poor, whether they are left to die, killed in inhumane ways, or kept in inadequate environments with little or no regard to their behavioural or environmental needs [22,23,34,68].

### 4.4. Additional Recommendations

Research on the impacts of trade on fish welfare is needed to make informed ethical judgements relating to policy decision-making (e.g., changes to how trade is conducted or prohibiting certain practices). Based on our findings that highlight the complexity and diversity of fish sentience, we recommend that if fish continue to be bred, traded, and caught, then the legislation governing these practices should be evaluated, improved, and enforced, taking fish sentience into full account. Where certain practices are already prohibited, such as shark finning for certain species [69] and electrical pulse fishing in the European Union in 2021 [70], these bans must be properly enforced to be effective.

Industries using fish, such as aquaculture, are growing exponentially [3], and some areas of welfare, such as slaughter practices, have benefitted from scientific attention [71,72,73]. Despite this, other areas are relatively disregarded, such as the millions of tonnes of wild-caught fish used for feeding farmed fish each year [21], and the welfare issues associated with their capture and death [34]. Fish are utilised in their trillions every year, so it is time that their sentience is properly recognised, and that practices and legislation affecting fish seek to safeguard fish welfare.

## 5. Conclusions

Our review has highlighted evidence of fish sentience in the scientific literature, but what does this mean in terms of the practices that impact the welfare of fish? According to the International Union for Conservation of Nature (IUCN), around a third of vertebrate species listed under the category ‘Use and Trade’ are fish [74], yet fish are rarely given much consideration in terms of legislative protection [23]. Fish are wild-caught and captive-bred for aquaculture, scientific research, traditional medicine, and the pet trade in vast numbers, yet little is known about how best to ensure their welfare and provide for their needs [75]. Furthermore, fish are a diverse group of animals, from seahorses to sharks, and what is considered acceptable by society is influenced by the type of fish in question. Shark finning, for example, is generally considered to be inhumane and criticised by the Western world as cruel, unnecessary, and wasteful, and there is legislation governing the practice, albeit widely considered to be ineffective [69]. However, practices in commercial fishing, such as leaving fish to die by asphyxiation, are commonly overlooked by society and in legislation, despite also being an inhumane practice [34].

Understanding more about the sentience of fish and publicising the scientific knowledge of fish sentience are crucial in changing both the public’s and industry’s attitudes and ultimately behaviour towards these animals. It is clear from our review that fish are already widely accepted as being capable of a range of sentient traits, yet practice and legislation do not always properly reflect this [23]. Targeted research that seeks to highlight the sentience of fish and how it relates to their welfare needs is therefore still needed.

## Supplementary Materials

The following supporting information can be downloaded at: https://www.mdpi.com/article/10.3390/ani12091182/s1, Supplementary File S1 and S2: Species and keywords [76,77,78].
