# Supplementary material for: A Kettle of Fish: A Review of the Scientific Literature for Evidence of Fish Sentience
Source: Animals (Basel). 2022 May 5;12(9):1182. doi: 10.3390/ani12091182 (PMC9100576; doi:10.3390/ani12091182)
Supplement: Supplementary file 1 [file animals-12-01182-s001.zip › Supplementary File S1.pdf]

Supplementary File S1. The keywords used in the study, including details of their source, valence and whether they returned suitable results.

	Keyword	Origin	Valence	Returned results
	Apprehension	**Plutchik (1981), Parrot (2001), Proctor *et al*. (2013)**	Negative	**No**
	Annoyance	**Plutchik (1981), Parrot (2001), HUMAINE (2006), Proctor *et al*. (2013)**	Negative	**No**
	Anticipation	**Plutchik (1981), Proctor *et al*. (2013)**	Neutral	**Yes**
	Anger	**Plutchik (1981), Parrot (2001), HUMAINE (2006), Proctor *et al*. (2013)**	Negative	**No**
	Arousal (emotional)	**Parrot (2001), Proctor *et al*. (2013)**	Neutral	**Yes**
	Agitation	**Parrot (2001), Proctor *et al*. (2013)**	Negative	**No**
	Anxiety	**Parrot (2001), HUMAINE (2006), Proctor *et al*. (2013)**	Negative	**Yes**
	Altruism	**Proctor *et al*. (2013)**	Positive	**Yes**
	Affective State	**Proctor *et al*. (2013)**	Neutral	**Yes**
	Contentment	**Parrot (2001), Proctor *et al*. (2013)**	Positive	**No**
	Conscious	**Proctor *et al*. (2013)**	Neutral	**No**
	Cognitive bias	**Lambert *et al.* (2019)**	Neutral	**No**
	Disgust	**Plutchik (1981), Parrot (2001), HUMAINE (2006), Proctor *et al*. (2013)**	Negative	**No**
	Dislike	**Parrot (2001), Proctor *et al*. (2013)**	Negative	**Yes**
	Depression	**Parrot (2001), Proctor *et al*. (2013)**	Negative	**Yes**
	Dismay	**Parrot (2001, Proctor *et al.* (2013)**	Negative	**No**
	Distress	**Parrot (2001), Proctor *et al*. (2013)**	Negative	**Yes**

	Excitement	**Parrot (2001), Proctor *et al*. (2013)**	Positive	**No**
	Eagerness	**Parrot (2001), Proctor *et al*. (2013)**	Positive	**Yes**
	Emotion	**Proctor *et al*. (2013)**	Neutral	**Yes**
**F**	Fear	**Plutchik (1981), Parrot (2001), HUMAINE (2006)**	Negative	**Yes**
	Frustration	**Plutchik (1981), Parrot (2001), HUMAINE (2006)**	Negative	**Yes**
**G**	Grief	**Plutchik (1981), Parrot (2001), Proctor *et al*. (2013)**	Negative	**No**
	Hostility	**Parrot (2001), Proctor *et al*. (2013)**	Negative	**Yes**
	Helplessness	**HUMAINE (2006), Proctor *et al*. (2013)**	Negative	**Yes**
	Intentionality	**New**	Neutral	**Yes**
**J**	Joy	**Parrot (2001), Plutchik (1981), Proctor *et al*. (2013)**	Positive	**No**
	Judgement bias	**Lambert *et al.* (2019)**	Neutral	**No**
	Nervousness	**Parrot (2001), Proctor *et al*. (2013)**	Negative	**No**
**O**	Optimism	**Parrot (2001), Plutchik (1981), Proctor *et al*. (2013)**	Positive	**No**
	Pleasure	**Parrot (2001), Proctor *et al*. (2013)**	Positive	**No**
	Panic	**Parrot (2001), Proctor *et al*. (2013)**	Negative	**Yes**
	Pessimism	**Proctor *et al*. (2013)**	Negative	**No**
	Play	**Proctor *et al*. (2013)**	Positive	**No**
	Pain	**Proctor *et al*. (2013)**	Negative	**Yes**
	Prosocial	**New**	Neutral	**No**
	Rationality	**Proctor *et al*. (2013)**	Neutral	**No**
**S**	Surprise	**Plutchik (1981), Proctor *et al*. (2013)**	Neutral	**No**

	Sentience	**Proctor *et al*. (2013)**	Neutral	**Yes**
	Suffering	**Parrot (2001), Proctor *et al*. (2013)**	Negative	**No**
	Stress	**Proctor *et al*. (2013)**	Negative	**Yes**
	Tenseness	**Parrot (2001), Proctor *et al*. (2013)**	Negative	**No**
	Valence	**Proctor *et al*. (2013)**	Neutral	**No**
